# Commemorating the Launch of the Section “Marine Toxins”

**DOI:** 10.3390/md24020073

**Published:** 2026-02-10

**Authors:** Andrew Turner, Panagiota Katikou

**Affiliations:** 1 Toxins and Contaminants Domain, Health and Welfare Division, CEFAS, Barrack Road, Weymouth, Dorset DT4 8UB, UK; 2 Directorate of Research, Innovation and Education, Ministry of Rural Development and Food, Hapsa & Karatasou 1, 54626 Thessaloniki, Greece

Marine toxins are natural compounds produced by a variety of marine organisms, including microalgae, bacteria, and macroalgae. These compounds have wide-ranging structural chemistries and biological activities. Many of the biological effects negatively impact health, including toxicity to humans and animals, as well as whole marine ecosystems. Toxins identified as hazards in the marine environment range from small polar compounds to high-molecular-weight lipophilic compounds. Toxicological effects are also wide-ranging, with some of these toxin groups resulting in human fatalities in the absence of proper risk management. Conversely, some of these compounds have shown promise for applications in medicine, biotechnology, and agriculture, highlighting the potential benefits that originate from aquatic toxins. This duality underlines the importance of marine toxins as both indicators of ecosystem stress and sources of bioactive compounds of practical interest.

The realities of the impacts of these toxins have resulted in the development of hazard monitoring and risk management strategies, together with well-developed regulatory programmes, across the world. Many of these toxins are known to bioaccumulate in the flesh of marine organisms that are consumed by humans as food products, notably bivalve molluscs, crustaceans, and fin fish. Over the past 20 years, many technological developments have taken place, empowering industry and regulatory authorities to develop monitoring approaches for assuring the safety of seafood products. Most notably, there have been significant developments in the move away from the use of live animal bioassays for food safety testing toward using instrumental chemical detection methods for official control testing of bivalves. Yet, in addition to well-recognised regulated toxins, other families of marine toxins that demonstrate toxicity effects in mammals and other organisms have emerged. Researchers and regulators around the world are working hard to develop approaches to monitoring such toxins, including approaches which are beneficial in their risk management. In addition, there is much work focused on the determination of the exact sources of such toxins, which include microalgae of wide-ranging ecologies, as well as multiple species of marine bacteria. In many cases, the full production mechanisms of toxin production are not yet fully understood; nor are the impacts of environmental changes on the prevalence and toxicity of toxin producers. The mammalian toxicity mechanisms for some emerging toxin hazards also require further elucidation.

The section “Marine Toxins” was instigated in June 2022 to provide a comprehensive publication platform for scientists working within multi-disciplinary fields linked to marine-derived toxins. As of January 2026, there have been 14 Special Issues and a total of 121 published manuscripts, highlighting the relevance of this section to aquatic science throughout the world. It provides a platform for researchers to share their latest findings on the isolation, analysis, characterization, biosynthesis, and biological activities of these bioactive molecules. It also includes work conducted by researchers focusing on the potential benefits to be gained from the incorporation of marine toxins into drugs of therapeutic benefit. In this way, this section explicitly connects fundamental toxin research with applied, regulatory, and public health perspectives.

The call for submissions for this Special Issue, “Commemorating the Launch of the section Marine Toxins”, opened in April 2023 and closed in March 2025. The final publication highlights and celebrates the importance of monitoring and studying these toxins, with a focus on new detection approaches, emerging hazards in new geographical regions, toxicology and the physiological effects of toxins in marine organisms and mammals, method refinements to improve toxin detection, the characterization of microalgal toxin producers, and the impact of environmental parameters on toxin-producing organisms. Together, these articles demonstrate the ongoing need to develop our shared understanding of the toxicological impacts of marine toxins on human, animal, and ecosystem health, as well as the importance of determining toxin production mechanisms and, ultimately, suitable risk monitoring approaches to mitigating these important hazards within our marine environments. Collectively, the contributions illustrate the breadth of marine toxin research, spanning molecular mechanisms, analytical innovation, ecological drivers, and implications for human and animal health.

The following section provides a brief overview of each of the eleven contributions to this Special Issue, together with links to the full published articles. These cover many of the most important marine toxin impacts, including domoic acid, saxitoxins, tetrodotoxins, ciguatoxins, and azaspiracids, as well as one paper on the toxicity of jellyfish venom.

***Contribution 1:***

Nutrient Deficiency Impact on the Cellular and Metabolic Responses of Saxitoxin-Producing *Alexandrium minutum*: A Transcriptomic Perspective. *Mar. Drugs* **2023**, *21*(9), 497; https://doi.org/10.3390/md21090497.

The article by Akbar et al. describes work conducted to understand the impacts of nutrient-deficient environments on the saxitoxin-producing dinoflagellate *Alexandrium minutum.* Whilst a great deal of information has been reported in the literature on toxin production by this species, the authors identified a need to assess the impacts of nitrogen and phosphorous depletion on the organism. Transcriptomic responses were assessed by the study team, resulting in the identification of specific differentially expressed genes for both nitrogen and phosphorous deficiency, with gene set enrichment analysis revealing enriched gene ontology terms associated with nutrient deficiency. This work was conducted using laboratory cultures, and it highlighted cellular and metabolic changes in the dinoflagellate, developing our understanding of the impacts of environmental changes on the ecophysiology of this toxin-producing organism.

***Contribution 2:***

Monthly Variation of Tetrodotoxin Levels in Pufferfish (*Lagocephalus sceleratus*) Caught from Antalya Bay, Mediterranean Sea. *Mar. Drugs*, **2023**, *21*(10), 527; https://doi.org/10.3390/md21100527.

Tetrodotoxins (TTXs) have long been associated with risks to human health following the consumption of pufferfish species, most notably in Japan following consumption of the pufferfish delicacy known as *Fugu.* Pufferfish are not limited to Asia, however, with invasive pufferfish species well recognized as spreading to other continents, notably into the Mediterranean Sea, where they impact both native species and human health. Consequently, there is a need to assess TTX concentrations in non-native pufferfish within European waters to assess likely impacts on human health for pufferfish consumers in the region. In this study, the authors conducted an assessment of TTX levels using high-resolution mass spectrometry, through the analysis of 110 pufferfish sampled monthly over a twelve-month period. Fish were dissected and different organs analysed, with quantitative analysis confirming the highest toxin concentrations in the gonads, followed by the liver and edible tissue, respectively. Seasonal variability was also assessed, along with differences in toxin levels between male and female fish. The results confirmed significant health risks for pufferfish consumers in the region.

***Contribution 3:***

Physiological Effects of Oxidative Stress Caused by Saxitoxin in the Nematode *Caenorhabditis elegans. Mar. Drugs* **2023**, *21*(10), 544; https://doi.org/10.3390/md21100544.

A high proportion of research work conducted on the potent marine neurotoxin saxitoxin (STX) relates to its impacts on mammalian health, most notably the health of human seafood consumers. Impacts on other animals such as birds and sea mammals are also well reported throughout the world. This study focusses instead on evaluating the toxicity of STX to nematodes, specifically *Caenorhabditis elegans*, a species of soil-dwelling roundworm. Worms were exposed to STX over a range of concentrations for 24 h and paralysis symptoms were observed but with the majority recovering. The team assessed multiple aspects of the nematode response, including analyses of lifespan, brood size, growth ability, reactive oxygen species, and adenosine triphosphate levels, together with the overexpression of green fluorescent protein. Declines were observed in the exposed worms, which, together with other exposure experiments, highlighted evidence of significant oxidative stress on *C. elegans* following STX exposure, indicating its potential use in ongoing assessment of invertebrate health following neurotoxin exposure.

***Contribution 4:***

Comparative Analysis of Tentacle Extract and Nematocyst Venom: Toxicity, Mechanism, and Potential Intervention in the Giant Jellyfish *Nemopilema nomurai*. *Mar. Drugs* **2024**, *22*(8), 362; https://doi.org/10.3390/md22080362.

This study reports the findings of Geng et al., who assessed the toxicity and mechanism of toxicity in tentacle extract (TE) and nematocyst venom extract (NV) from the giant jellyfish *Nemopilema nomurai.* This scyphozoan is a major species of concern in China, Korea, and Japan and is widely associated with stings in human bathers, although the full toxicity effects and biological actions of the venoms have yet to be fully elucidated. In this study, the authors recognized the need to assess and compare the effects of venoms from TE and NV, investigating venom composition and toxicity. Multiple assessments were conducted, which showed the impacts of these important venoms, with the authors furthermore assessing the methodological impacts of extraction methods, together with the potential promising application of an antidote.

***Contribution 5:***

Morphological, Toxicological, and Biochemical Characterization of Two Species of *Gambierdiscus* from Bahía de La Paz, Gulf of California. *Mar. Drugs* **2024**, *22*(9), 422; https://doi.org/10.3390/md22090422.

Ciguatera Fish Poisoning (CFP) is known to be the most prevalent marine toxin-triggered seafood poisoning syndrome in the world, with reports indicating that more than 50,000 people are affected annually, with the majority of incidents reported in tropical and sub-tropical regions of the Pacific, Indian, and Caribbean Oceans. The ciguatoxins and sometimes maitotoxins that cause these human illnesses are produced by a range of benthic dinoflagellates of the genera *Gambierdiscus* and *Fukuyoa.* Multiple *Gambierdiscus* species have been previously identified as ciguatoxin (CTX) producers, with this list expanding as more investigations are conducted. In this study, the authors investigated new isolates of two species (identified as *G.* cf. *caribaeus* and *G.* cf. *carpenteri*) from the southern Gulf of California. The assessments included morphological characterization using light microscopy and scanning electron microscopy, pigment and amino acid analysis and toxicity through a receptor binding assay, and the application of several biological assays. CTX-like toxicity was confirmed in all strains, showing potential risks to human fish consumers in the region from ciguatera poisoning.

***Contribution 6:***

Marine Algal Toxins and Public Health: Insights from Shellfish and Fish, the Main Biological Vectors. *Mar. Drugs* **2024**, *22*(11), 510; https://doi.org/10.3390/md22110510.

The impacts of marine toxins have long been recognised on the health of the human seafood consumer, with reports of poisonings stretching back several hundred years in some instances. Similarly, there are multiple reports linking the presence of certain pelagic and benthic microalgal species to both toxin production in marine waters and the subsequent bioaccumulation of toxins in wide-ranging marine organisms, most notably, invertebrates such as bivalve molluscs. In this paper, the authors present a review of the current knowledge regarding public health impacts of marine biotoxins, including reports of exposure routes from both shellfish and fish, which are shown to be the most common biological vectors for human poisonings worldwide. Changes in HAB/toxin occurrence are also described, as well as the requirements for a great understanding of the toxicological impacts of major seafood toxin poisoning syndromes, specifically, DSP, PSP, ASP, NSP, and CFP.

***Contribution 7:***

Optimization of the Extraction Protocol for Pacific Ciguatoxins from Marine Products Prior to Analysis Using the Neuroblastoma Cell-Based Assay. *Mar. Drugs* **2025**, *23*(1), 42; https://doi.org/10.3390/md23010042.

Ciguatera Poisoning (CP) is widely recognised as having a huge impact on human health globally, most notably in developing countries and regions. The impacts on health have been reported most commonly in countries on the Pacific Ocean, where ciguatoxins (CTXs) produced by a range of benthic dinoflagellates are responsible for bioaccumulation in carnivorous fish species, which are sometimes the only source of protein for local populations. Risk management in these fish is notoriously complex, given the high toxicity originating from even very low toxin concentrations, as well as the highly variable chemistries of wide-ranging CTX analogues, with variations reported between different regions and different fish species. Whilst multiple analytical methods have been published for Pacific CTX (P-CTX) determination, the best setup for many of them remains unknown, which is understandable, given the absence of certified reference standards for the key toxin analogues. Consequently, the authors of this study sought to better understand the performance of these techniques through the evaluation of six extraction and purification protocols, in order to assess method recovery following analysis of fish tissues spiked with P-CTX standard. Better method performance was reported, together with recommendations for future improvements in both cell toxicity and mass spectrometric analysis.

***Contribution 8:***

The Toxic Effects of Environmental Domoic Acid Exposure on Humans and Marine Wildlife. *Mar. Drugs* **2025**, *23*(2), 61; https://doi.org/10.3390/md23020061.

One of the most widely cited examples of marine toxin-related human poisonings from seafood consumption was in Canada in the 1980s, where sickness resulted from consumption of molluscs containing domoic acid (DA). This toxin is produced naturally by some species of the marine diatom *Pseudo-nitzchia* and some red macroalgal species and results in the poisoning syndrome termed Amnesic Shellfish Poisoning (ASP). This toxin not only is a significant and regulated threat to human health around the world but has been linked to multiple incidents of animal health impacts in a large variety of organisms such as sea mammals and seabirds. The authors of this paper sought to review these impacts with a focus on both humans and animals, providing an overview of DA production, toxicity including clinical signs and medical treatment approaches, toxicokinetic properties, susceptibility to DA toxicosis and detection methods used for risk management purposes. Overall, the review provides a timely reminder of all the important impacts that this low-molecular-weight toxin has on both human and animal health throughout the world.

***Contribution 9:***

First Confirmed Occurrence of Ciguatera Poisoning in the UK from Imported Pinjalo Snapper (*Pinjalo pinjalo*). *Mar. Drugs* **2025**, *23*(2), 67; https://doi.org/10.3390/md23020067.

Ciguatera Fish Poisoning (CFP) is traditionally associated with outbreaks in tropical and sub-tropical regions of the world, with poisonings associated with human consumption of carnivorous fish species which have accumulated ciguatoxins produced by various benthic dinoflagellate species. However, over the last decade, several reports have been published describing human poisonings in fish consumers from temperate latitudes, where the causative products were fish imported into these regions from Pacific or Indian ocean territories. This study presents evidence for the first recorded outbreak of CFP in the UK, which occurred following the import of fish steaks labelled as Red Snapper (*Lutjanus bohar*) from the Indian Ocean. Clinical symptoms were recorded in three affected people, with the leftover fish tissues subsequently assessed for CTX presence using both cytotoxicity and mass spectrometry tools. The authors used a neuroblastoma assay to confirm sodium channel activity consistent with CFP-like activity, with mass spectrometry evidencing the presence of tri- and di-hydroxylated P-CTX 3C congeners. Together the evidence highlighted significant risks from consumption of imported fish fillets in geographical regions where CFP incidents are unexpected.

***Contribution 10:***

Comparative Analysis of Intestinal Microbiota Between Tetrodotoxin-Containing and Tetrodotoxin-Free *Takifugu rubripes. Mar. Drugs* **2025**, *23*(4), 140; https://doi.org/10.3390/md23040140.

The well-known presence of TTXs in pufferfish has long been reported as potentially relating to symbiotic bacteria held within the intestine of the host pufferfish, even though the specific bacterial species and the processes involved in this production and accumulation are yet to be elucidated. Consequently, in this study, the authors report on an experiment where the intestinal microbiota was analysed in both toxic and non-toxic pufferfish of the species *Takifugu rubrupes.* The results revealed that significant differences existed between the toxic and non-toxic fish, with key genera identified. As a consequence, the authors proposed that future toxin-screening approaches might include the use of symbiotic bacteria screening for biomarker assessment for food safety in pufferfish.

***Contribution 11:***

High-Affinity Aptamers and Their Specificity for Azaspiracid-2 Using Capture-SELEX. *Mar. Drugs* **2025**, *23*(5), 183; https://doi.org/10.3390/md23050183.

Alongside the vast development in recent decades of state-of-the-art laboratory tools for the detection and quantitation of regulated and non-regulated marine toxins in seafood products, there is the need for Food Business Operators to conduct End-Product Testing (EPT) of shellfish batches for marine toxin detection, as part of their hazard monitoring processes. Multiple commercial EPT assays are available around the world for the majority of regulated toxin families, but to date, no such tools have been commercialised for the azaspiracids: toxins that are incorporated into the lipophilic toxin family. This study reports on the development of approaches focussed on finding an aptamer-based solution for AZA detection, specifically by screening aptamers specific to the AZA analogue, AZA-2, in tandem with an in silico determination of aptamer binding sites and stability of aptamer–toxin complexes. As a consequence, two aptamers were obtained, dissociation constants defined, and the application to a new detection method for AZA detection discussed.

